# Addition of silver nanoparticles to universal adhesive: In vitro effect on biofilm and shear bond strength

**DOI:** 10.1590/0103-644020256255

**Published:** 2025-09-19

**Authors:** Jaqueline Costa Favaro, Omar Geha, Talita Nicaretta Canavarros, Alessandra Nascimento, Murilo Baena Lopes, Ricardo Danil Guiraldo, Sandrine Bittencourt Berger

**Affiliations:** 1 Department of Restorative Dentistry, University Anhanguera-Uniderp, Department of Restorative Dentistry, Campo Grande, Mato Grosso do Sul, Brazil.; 2 Department of Restorative Dentistry, University of North Parana, Londrina, PR, Brazil.; 3 University of Cuiabá, Department of Dentistry, Cuiabá, Mato Grosso, Brazil.

**Keywords:** Silver nanoparticle, Self-etch adhesive, Antibacterial adhesives, Shear strength. Biofilm

## Abstract

Silver nanoparticles (AgNPs) have been incorporated into dental materials at low concentrations to provide antibacterial action without compromising mechanical properties. This in vitro study evaluated the antibiofilm effect and bond strength of an experimental universal adhesive with AgNPs (EAg). *Streptococcus mutans* biofilm was induced by incubating samples with 0.01% and 0.02% AgNPs, based on a pilot study, compared to a control (experimental without AgNPs, ES) in a 20% sucrose medium. This was followed by sonication and counting of viable cells after 1 and 7 days (n = 9). Enamel and dentine bovine micro shear bond strength test (μ-SBS) was performed (n=10) with EAg0.01% and controls (ES and the commercial OPT (Optbond Universal; Kerr). μ-SBS data of enamel and dentin were evaluated for normality and homogeneity by Shapiro-Wilk and Levene, respectively, resulting in normality. Therefore, they were subjected to ANOVA. The failure type was evaluated using a stereoscopic magnifying glass at '40 and categorized as adhesive, cohesive, and mixed failure. The 0.01% concentration demonstrated the antibiofilm effect at the lowest AgNP concentration and was selected for the μ-SBS test. For μ-SBS ANOVA, there were no statistically significant differences between experimental and commercial adhesives (p<0.05). Evaluation of failure mode showed a predominance of adhesive failure on both substrates for all adhesives. The EAg exhibited antibiofilm activity with adhesive performance statistically similar to that of the commercial adhesive. Experimental universal adhesive containing silver nanoparticles showed antibacterial activity without compromising μ-SBS to enamel and dentin

## Resumo

Nanopartículas de prata (AgNPs) têm sido utilizadas em materiais odontológicos, em baixas concentrações, para proporcionar ação antibacteriana, sem alterar as propriedades mecânicas. Este estudo in vitro avaliou o efeito antibiofilme e a resistência de união de um adesivo universal experimental com AgNPs (EAg). O biofilme de *Streptococcus mutans* foi induzido pela incubação de amostras com 0,01 e 0,02% de AgNPs, com base em estudo piloto, comparados ao controle, experimental sem AgNPs (ES), em meio de sacarose a 20%, seguido de sonicação e contagem de células viáveis após 1 e 7 dias (n=9). O teste de resistência ao microcisalhamento (μ-SBS) foi realizado em esmalte e dentina bovinos (n=10) com EAg0,01% e controles (ES e o comercial OPT (Optbond Universal; Kerr). Os dados de μ-SB em esmalte e dentina foram avaliados quanto à normalidade e homogeneidade por Shapiro-Wilk e Levene, respectivamente, resultando em normalidade. Portanto, eles foram submetidos à ANOVA. O tipo de fratura foi avaliado com lupa estereoscópica (10x - BelMicroimage Analyzer; Bel Photonics) e categorizado em falha adesiva, coesiva e mista. A concentração de 0,01% apresentou efeito antibiofilme com menor concentração de AgNPs e foi selecionada para o ensaio μ-SBS. Para μ-SBS ANOVA não apresentou diferenças estatisticamente significativas entre os adesivos experimentais e comerciais (p<0,05). A avaliação do índice restante revelou predomínio de falha adesiva em ambos os substratos para todos os adesivos. EAg apresentou atividade antibiofilme com desempenho adesivo estatisticamente semelhante ao adesivo comercial.

**Figure:**
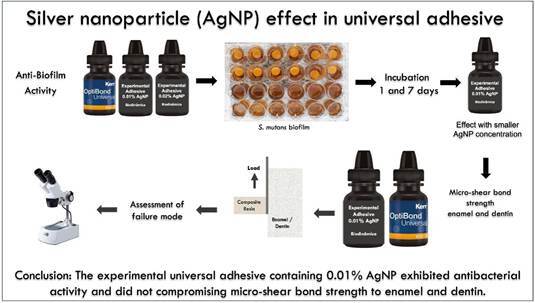

## Introduction

Adhesive systems and resin composites provide optimal functional and esthetic results while preserving healthy tooth structure. ^1^ due to their excellent aesthetics and direct-filling capabilities ^2^. The integrity of resin composite restorations relies on the formation of hybrid layers ^1^. However, adhesive failure is the primary factor reducing their longevity ^3^. Microleakage and gap formation in this weakened interface provide a practical pathway for the invasion of oral plaque biofilms and the development of secondary caries around the tooth-restoration margins ^1^ ^,^ ^3^. *Streptococcus mutans* has been identified as the primary cariogenic pathogen due to its ability to form biofilms, its acidogenicity, and its aciduricity. Consequently, it can significantly damage the adhesive interface ^3^ ^,^ ^4^ and also degrade resin composites and adhesives ^3^ ^,^ ^5^ ^).^

Silver nanoparticles are highly effective antimicrobial agents, with their action being more pronounced than that of ionic silver. They have a greater surface area available to interact with the microorganism, and their antibacterial activity is related to the size of their particles; that is, their antimicrobial effect increases with decreasing particle size ^6^. The antimicrobial mechanism of silver nanoparticles occurs due to their ability to penetrate the bacterial cell wall, damaging it through direct and indirect lipid peroxidation, thus interrupting cell processes, such as DNA replication and cellular respiration, at low concentrations (1.6-10). Ag NPs can block the synthesis of bacterial exopolysaccharides and then biofilm. The ability of biofilm inhibition by Ag NPs may be due to the existence of water channels throughout the biofilm. They can enter biofilms and inhibit biofilm development by suppressing gene expression. These ions are internalized and prevent the penetration of amines, thiols, or carboxylates. Ag has a strong inhibitory effect on the strong biofilms ^11^. Furthermore, silver nanoparticles are non-toxic to both animals and human cells ^1^ ^,^ ^3^ ^,^ ^5^ ^,^ ^4^ ^,^ ^5^ ^,^ ^6^ ^,^ ^7^ ^,^ ^12^ ^).^

Studies have tested materials with silver nanoparticles to reduce microbial adhesion to restoratives, presenting promising results ^1^ ^,^ ^2^ ^,^ ^3^ ^,^ ^5^ ^,^ ^13^ ^).^ These results have been achieved without compromising the degradation of resin-dentin surfaces while also improving the mechanical properties ^2^ ^,^ ^3^ and enhancing the microshear bond strength of adhesives in adhesive systems ^3^. The development of adhesives with antimicrobial activity could prevent the formation of recurrent caries at the tooth-restoration interface and, consequently, failure of the resin restorations ^1^ ^,^ ^2^ ^,^ ^3^ ^,^ ^7^ ^,^ ^8^ ^).^

.Despite the promising antibacterial properties of silver nanoparticles (AgNPs) and their potential to enhance the longevity of adhesive restorations, there is limited evidence on the impact of incorporating AgNPs into universal dental adhesives on both biofilm inhibition and bond strength to enamel and dentin. Therefore, the objectives of this study were to develop a universal dental adhesive containing silver nanoparticles in low concentration, determine its antibacterial activity against *Streptococcus mutans* (*S. mutans*) biofilm, and investigate the effect of the micro-shear bond strength (μ-SBS) on enamel and dentin surface. The null hypothesis tested is that the use of a universal dental adhesive containing silver nanoparticles in low concentration does not affect enamel and dentine μ-SBS values, with antibacterial effects against all *S. mutans* biofilm.

## Material and methods

In this study, a commercially available single-component self-etching adhesive, OPT (Optibond Universal; Kerr Corporation, California, USA), and three experimental single-component self-etching adhesives were tested. The experimental adhesives were manufactured by Biodinâmica (Ibiporã, Paraná, Brazil). They contained spherical silver nanoparticles with an average size of 30 nm, stabilized with ethylene glycol and polyvinylpyrrolidone, obtained through chemical coprecipitation reactions with a pH range of 3 to 5, as characterized and stabilized in a previous study ^6^. For all analyses, the evaluator was blinded; however, due to the material's color, it was not possible to blind the operator.

### Experimental Adhesives Description

The silver nanoparticle concentrations were based on a pilot study that found an antibacterial ability with 0.01% silver nanoparticles. Therefore, the silver nanoparticles were incorporated into experimental adhesives at the following proportions: 0.01% (EAg0.01), 0.02% (EAg0.02), and 0.0% (ES), which was regarded as the positive control group. The composition of the materials is described in Table 1.

**Table 1:** Description, and composition of adhesives used in the study.

Adhesive	Description	Composition
OPT - Optibond Universal (Kerr)	Single-component self-etching adhesive	Ethanol, HEMA, GPDM, pyrogenic (Fumed) Amorphous Silica, Alkali fluorosilicates (Na)
ES (Biodinâmica)	Experimental single-component self-etching adhesive without silver nanoparticles	BisGMA, HEMA, GPDM, ethyl alcohol, camphorquinone and water
EAg 0.01 (Biodinâmica)	Exeperimental single-component self-etching adhesive with silver nanoparticles	0.01% silver nanoparticles, BisGMA, HEMA, GPDM, ethyl alcohol, camphorquinone and water
EAg 0.02 (Biodinâmica)	Exeperimental single-component self-etching adhesive with silver nanoparticles	0.02% silver nanoparticles, BisGMA, HEMA, GPDM, ethyl alcohol, camphorquinone and water

### Dental adhesive disk preparation

Disks for antibacterial tests were fabricated following the previous study ^7^. The adhesive disks are obtained using a metal mold (diameter 6 mm, thickness 1 mm) with a glass slide underneath. Each adhesive was dripped onto the metal mold it was filled. A polyester strip was positioned and cured for the 20s on each side using an LED curing unit (Radii-cal, SDI, Bayswater, VIC, Australia) at a light intensity of 1,200 mW/cm^2^), A radiometer (Demetron L.E.D. Radiometer, Kerr Sybron Dental Specialties, Middleton, USA) was used to check the light intensity for every five specimens. Then, the disks were removed from the rings without damage. All samples were stirred and cleaned by sonicating for 5 min to remove any uncured monomers. The specimens were stored in sterile water for 24 hours and then dried and sterilized at 121 °C under a pressure of 1 atm for 15 minutes (Cristófoli, Paraná, Brazil) for antibiofilm investigation.

### Biofilm formation

Assays were based on the criteria described by the National Committee for Clinical Laboratory Standards for bacteria, M07-A10 ^14^. All assays were performed in triplicate and three times to ensure reproducibility ^2^ ^,^ ^3^ ^,^ ^5^.

*S. mutans* from American Type Culture Collection (ATCC) 35668 was used. The microorganism strains were cultured aerobically in a brain heart infusion (BHI) medium, modified with 20% sucrose. The culture was incubated at 37 °C for 24 h. After incubation, a bacterial suspension was prepared with standardized absorbance. The culture was centrifuged at 3000 rpm for 20 minutes (Excelsa II centrifuge, model 206-BL; FANEM, São Paulo, SP, Brazil). Next, the supernatant was discarded, and the precipitated biomass was resuspended in phosphate-buffered saline (PBS) until it reached an absorbance corresponding to McFarland scale 0.5 (number of cells in the order of 108 colony-forming units -CFU/mL) ^5^.

The adhesive disks of each group were placed into each well of 24-well plate filled with 1 mL of 20% sucrose BHI and 100 µl of standardized S. mutans suspension in PBS. Biofilm was grown on nine discs for each time point (1 and 7 days) from all adhesive groups. To induce biofilm formation, the plates were incubated in 5% CO2 at 37°C, and the time points chosen for sonication of biofilms were 1 and 7 days ^5^.

### Biofilm Quantification

After each incubation period (1 and 7 days), the discs of all groups were washed three times with sterile PBS to remove non-adherent bacteria and then placed in tubes containing 10 mL of PBS. These tubes were shaken for 1 minute and then placed in an ultrasonic tank for 8 minutes (Digital Ultrasonic Cleaner, with a cleaning power of 160 W; Kondortech, São Carlos, SP, Brazil). This procedure causes the biofilm disaggregation and releases the bacterial cells adherent to the discs. The viable cells in the resulting suspension were quantified after disaggregation by counting colony-forming units (CFU). Five dilutions were made from this initial suspension. In the first step, 100 μL of the initial suspension was inoculated into 900 μL of PBS (10-1 dilution), resulting in a final dilution of 10-5. From each dilution, 100 μL was pipetted onto MRS agar on a Petri dish (90 x 15 mL), and this inoculum was spread over the surface with a Drigalsky handle. The Petri dishes with S. mutans were incubated directly in the oven at 37°C for 48 h. After incubation, dishes with 30-300 colonies were selected for counting. This number was multiplied by the dilution factor and then by 10 to obtain the number of CFU present in 1 mL of the initial suspension. The table was structured with the CFU number per mL ^3^.

### Micro-shear bond strength (μ-SBS) 

Forty bovine incisors, enamel defects free, were used in this study. The teeth’s crowns were cleaned and stored in 0.5% chloramine T solution for 7 days; next, they were stored in distilled water at 4°C until they were used in the experiment. The crowns were separated from the roots, sectioning the teeth 1 mm apical to the cement enamel junction using a diamond disc. The crown portions were sectioned longitudinally into buccal and lingual halves. Enamel specimens were collected from the vestibular surface teeth (7 mm width × 4 mm length × 7 mm height). The enamel fragments were fixed with acrylic resin (JET, Clássico, São Paulo, SP, Brazil) in PVC tube rings (Odeme Dental Research, Luzerna, SC, Brazil). Enamel surfaces were abraded with 400, 600, 1,000, 1,200, and 1,500-grit silicon carbide paper and polished with abrasive paper and 1-µm diamond paste in electric polisher (APL4 Arotec S/A Ind. e Comércio, Cotia, SP, Brazil). For the dentin, the sectioned halves were placed in PVC tube rings filled with self-curing acrylic resin until the resin had been set. Dentin was exposed on the cervical one-third of the lingual surfaces of the teeth, and the surface was wet polished with 600-grit silicon carbide paper to obtain a flat surface.

Samples were then subjected to an ultrasonic bath in deionized water for 10 minutes (Model Ultrasonic Cleaner, Odontobras, Ribeirão Preto, SP, Brazil) to remove debris. Prepared specimens were examined under a stereomicroscope (Model Bel Photonics STM Pro, Bel Microimage Analyzer, Bel Photonics, Monza, Italy) at ×40 magnification to confirm the absence of cracks or other surface defects to enamel specimens and to ensure that no remnants of enamel were present on dentin specimens. Specimens were stored in deionized water until use to prevent dehydration.

Specimens were randomly categorized into one experimental universal adhesive with silver nanoparticles (EAg0.01 due to the anti-biofilm effect with minor nanoparticles concentration (6,7) and two control groups (ES and OPT). The sample size per group was calculated based on a pilot study, which found that 10 samples per group were required to obtain a power test of 80%, a standard deviation of 2.0 MPa, and a difference between means of 3 MPa.

### Adhesive procedures

Thirty-seven percent (37%) phosphoric acidic (Dentsply, Petropolis, RJ, Brazil) was applied on the flat enamel surface for 15 seconds; next, samples were washed for an additional 30 seconds and air-dried for 5 seconds. The bonding agents were actively applied with the aid of a brush, and the solvent was evaporated with air-jet on all specimens (enamel and dentin); the previous step was redone, and light-curing was carried out by using an LED curing unit (Radii-cal, SDI). Subsequently, three Tygon matrices (TYG-030, Saint-Gobain Performance Plastic, Miami Lakes, FL, USA) - 0.75 mm diameter and 1 mm height - were positioned on each sample using a clinical clamp. Resin composite (Filtek Z350, 3M ESPE, MN, USA) was applied to the matrix in a single increment with the aid of a calcium hydroxide applicator and light-cured for 40 seconds (Radii-cal, SDI) to produce resin composite cylinders. The samples were stored at 37 °C for 24 hours until bond strength measurements.

### μ-SBS

The Tygon matrix was carefully removed 24 hours after the adhesive procedure. The excess of resin composite and bonding agents in the enamel was removed with scalpel blade No. 11. Each resin cylinder was individually wrapped in steel wire (0.2-mm diameter), which was fixed to the shear device coupled to a universal test machine (EMIC DL 2000, Equipment and Testing Systems, EMIC, PR, Brazil). The μ-SBS test was performed at a speed of 0.5 mm/min until a fracture was produced. The shear strength value was transformed into megapascal (MPa) by using the load value indicated at fracture time (in Newtons) and divided by the inner surface area of the cylinder.

### Assessment of failure modes

All samples were observed under a stereomicroscope (Bel Microimage Analyser, Bel Photonics) at ×40 magnification to identify failure patterns. Failure modes were classified into the following three groups: adhesive (lack of adhesion), cohesive (tooth substrate or resin composite failure), or mixed (adhesive and cohesive failures).

Statistical Analysis

The data of viability S. mutans were tabulated, and their normality was assessed through the Shapiro-Wilk test. Afterward, a One-Way ANOVA followed by the Tukey test was used at a 5% significance level. The data of μ-SBS were tabulated, and the normality and homoscedasticity of all data were assessed using the Shapiro-Wilk and Levene tests, respectively, in Minitab 16 for Windows 8 software (Minitab Inc., State College, Pennsylvania, USA). Data presented normal distribution (p > 0.05) and homoscedasticity (p > 0.05). Thus, a one-way analysis of variance was applied, followed by an ANOVA test at a 5% significance level.

## Results

### Antibacterial effects

The results of the antibacterial efficacy of silver nanoparticles in dental adhesive against 1and 7 days *S. mutans* biofilms grown on the specimens' surfaces of experimental adhesive ES, EAg0.01, and EAg0.02 were determined using viable colony counts and are presented in Table 2 of mean and standard deviation values (CFU/mL). The results presented indicate that independently of the experimental groups tested (EAg0.01 and EAg0.02) or periods considered displayed lower viability levels when compared to biofilms about the control group (ES) where biofilms were grown. It can also be observed that EAg0.01 and EAg0.02 presented no statistical difference in antimicrobial activity with viability *S. mutans*. EAg0.01 was selected for the adhesion tests because it presented an antimicrobial effect with a minor AgNP concentration.

**Table 2:** Viable *Streptococcus mutans* cells expressed as colony forming units per mL of culture medium (CFU/mL) of experimental and control group (ES).

Treatment	Mean ( SD
ES	54.1 (17,9) A
Eg0.01	2.75 (4.83) B
Eg0.02	2.22 (1.96) B

Data are presented as means ± standard deviations. Values with different superscript letters were statistically different according to the Tukey test (p < 0.05).

### μ-SBS

**Figure 1:**
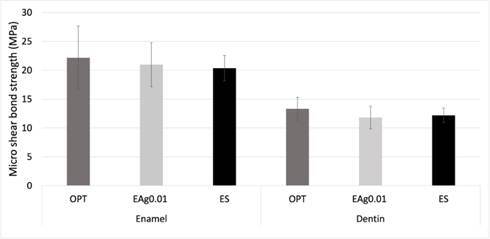
Mean of μ-SBS values (in MPa) of enamel and dentin according to groups

For μ-SBS, both substrates showed normal homogeneity (enamel, p = 0.45 and p =0.25; Dentin, p = 0.38 and p =0.25). Thus, the data were submitted to ANOVA, which did not reveal a statistically significant difference between the groups (enamel, p = 0.72; dentin, p = 0.37), as shown in Figure 1.

### Failure modes

**Figure 2:**
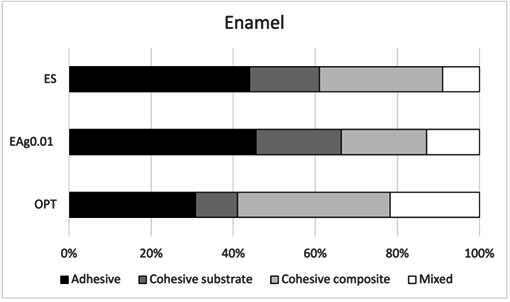
Failure modes of the adhesives bonded to the enamel from each group.

**Figure 3:**
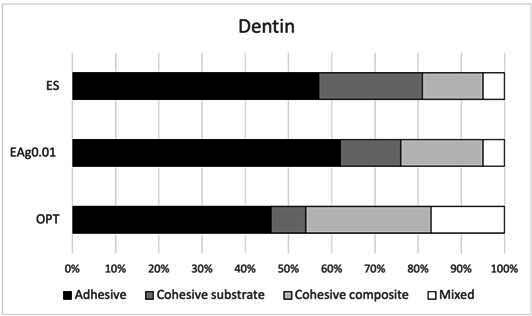
Failure modes of the adhesives bonded to the dentin from each group.

Figure 2 depicts the failure modes of the adhesives bonded to the enamel from each group. Adhesive failure was the prevalent fracture mode observed in ES and EAg0.01 groups, and it was followed by mixed and cohesive failures, respectively. The OPT group presented prevalent cohesive failures, followed by adhesive and mixed failures, respectively. Figure 3 depicts the failure modes of the adhesives bonded to the dentin from each group. Adhesive failure was the prevalent fracture mode that occurred most frequently at the interface on the dentin substrate for all adhesives tested.

## Discussion

As seen in the present in vitro study, the use of universal dental adhesive containing silver nanoparticles in low concentration (0.01%) did not affect μ-SBS values and presented antibacterial effects against *S. mutans* biofilm. The null hypothesis, that there is no difference in adhesion between the universal adhesives tested (OPT and EAg0.01) and that EAg0.01 is effective for *S. mutans* biofilm prevention, cannot be rejected. Indeed, silver nanoparticles have attracted considerable attention for their application in dental materials due to their antimicrobial effects and biocompatibility ^1^ ^,^ ^2^ ^,^ ^3^ ^,^ ^4^ ^,^ ^5^, ^6^, ^7^ ^,^ ^12^, which could, therefore, be very beneficial in avoiding restorative materials failures. Dental nanomaterials have potential for the future but currently do not always exhibit superior properties to commercial ones ^15^. Therefore, studies with new preventative dental products are welcome in dental practice.

In this study, several concentrations were tested in a pilot study to determine the optimal concentration(s) that resulted in better antibacterial properties without compromising μ-SBS compared to an available adhesive system. The anti-biofilm test showed that EAg0.01 was able to inactivate 100% growth of *S. mutans* at a low concentration of silver nanoparticles. This result may be related to the nanometric size of the silver particles used in the experimental solution (7-30 nm). Nanometric particles have a larger surface area available to interact with microorganisms ^6^ ^,^ ^11^ ^,^ ^16^ ^,^ ^17^ ^,^ ^18^ ^,^ ^19^ ^,^ ^20^, and they have a specific action mechanism that makes them a potent antimicrobial agent ^1^ ^,^ ^6^ ^,^ ^7^ ^,^ ^8^ ^,^ ^9^ ^,^ ^19^. The result found here, using a lower concentration of silver nanoparticles, corroborates with previous studies in which lower concentrations were used for its antibacterial effects and good biocompatibility ^1^ ^,^ ^3^ ^,^ ^6^ ^,^ ^7^ ^,^ ^8^ ^,^ ^9^ ^,^ ^21^. Smaller AgNPs can release more silver ions and have a greater surface area, increasing their antimicrobial effect ^6^ ^,^ ^18^.

An adhesive with antibiofilm activity is clinically significant, as incorporating antibacterial agents into adhesives can help combat biofilms and recurrent caries at the tooth-composite interface. Residual bacteria may persist within the prepared cavity, and microleakage over time can facilitate bacterial infiltration at the tooth-restoration margins, leading to secondary caries ^1^ ^,^ ^2^. Since the treatment of dental caries involves the removal of infected tissue and restoration with polymer-based materials, an imperfect bond between the tooth and the restoration can contribute to recurrent caries due to bacterial microleakage. Many polymer restorations require replacement within a few years because of persistent marginal gaps ^3^ ^,^ ^8^. Additionally, cavity preparation does not always eradicate bacteria within dentinal tubules and the smear layer, allowing them to survive, produce toxins, and potentially lead to restoration failure ^7^. In this context, AgNPs play a crucial role in disrupting bacterial biofilms and inhibiting the proliferation of cariogenic microorganisms at the adhesive interface, thereby enhancing the longevity and success of restorative treatments.

The μ-SBS test was used to measure bond strength to bovine dental enamel and dentin, and the results showed no statistically significant differences between experimental and commercial adhesives when comparing each substrate. Studies have tested different concentrations of silver nanoparticles and found that silver nanoparticles at low concentrations do not affect μ-SBS ^3^ ^,^ ^21^ ^,^ ^22^. No changes in the mechanical properties were probably because the silver nanoparticles were incorporated in the adhesive and could be photopolymerized ^3^. To obtain bond strength benefits, the adhesive must uniformly penetrate the collagen system of dentin and be effectively polymerized. In dentin, this adhesion stability could be explained because the functional monomers in self-etch adhesives can chemically interact with hydroxyapatite within a clinically acceptable time, and this chemical interaction could be enhanced by possible infiltration of silver nanoparticles into dentinal tubules and perhaps provide better resistance to degradation by preventing micro- and nanoleakage. ^22^. Then, the performance may have been due to silver nanoparticles being able to infiltrate the dentinal tubules due to their size ^3^. However, in enamel surfaces, the effect of etching on cavity surfaces has an important role in producing high bond strength ^22^.

The failure pattern analysis indicated the prevalence of adhesive failures over cohesive or mixed failures, which indicates that the bond strength values observed in the micro-shear test were obtained from the adhesive interface (dental structure and resin composite) ^23^. However, for the OP group in enamel, cohesive failures were prevalent. Fracture analysis is a crucial factor to consider when interpreting bond strength results. The failure pattern provides insight not only into the reliability of stress distribution during testing but also into the weakest point within the dentin-adhesive interface. Cohesive failures may indicate that the substrate or resin composite was overstressed during the test, thus causing its failure prior to that of the interface itself ^23^; this may be due to greater interaction between the adhesive and the substrate, resulting in a more durable bond. These results should be confirmed by studying the longevity of the bond strength.

Considering the limitations of this study that measured the antimicrobial effect and the bond strength for a short time, incorporating silver nanoparticles into the universal adhesive seems to be a promising way to obtain a strong bond, less subjected to the deleterious effects of bacteria without affecting cell viability comparing with a universal adhesive without silver nanoparticles added. More studies should be conducted to investigate the long-term durability of adhesives and their antibacterial characteristics. Furthermore, the results of this study were developed *in vitro*. Therefore, clinical investigations are required to establish these findings and provide clinical recommendations.

## Conclusion

The experimental universal adhesive containing silver nanoparticles exhibited antibacterial activity activities and did not without compromising micro-shear bond strength to enamel and dentin.
